# Epidemiological Surveillance of Chagas Disease in Rural Communities of a Municipality in Piauí, Brazil

**DOI:** 10.3390/life15121803

**Published:** 2025-11-25

**Authors:** Filipe Melo da Silva, Raphael de Lucena Banaggia, Giovana Marinho Arbusti, Stefanny Nunes Fidelis, Henrique Previtalli-Silva, Lucas Almeida Zangirolami, Flávia de Oliveira Cardoso, Katia da Silva Calabrese

**Affiliations:** 1 Laboratório de Protozoologia, Instituto Oswaldo Cruz, Fiocruz, Av. Brasil, 4.365, Manguinhos, Rio de Janeiro 21040360, Brazil; filipemelotkd@gmail.com (F.M.d.S.); raphaelbanaggia@gmail.com (R.d.L.B.); giovana.ma1@hotmail.com (G.M.A.); stenfw.7@gmail.com (S.N.F.); henriqueprevitalli89@gmail.com (H.P.-S.); lucas.zangirolami1@gmail.com (L.A.Z.); 2 Programa de Pós-Graduação em Medicina Tropical, Instituto Oswaldo Cruz, Fiocruz, Rua Magalhães Filho, 519, Centro/Norte, Teresina 64000128, Brazil; 3 Programa de Pós-Graduação em Biologia Parasitária, Instituto Oswaldo Cruz, Fiocruz, Av. Brasil, 4.365, Manguinhos, Rio de Janeiro 21040360, Brazil

**Keywords:** chagas disease, serology, epidemiology, diagnosis

## Abstract

Chagas disease, caused by *Trypanosoma cruzi*, remains a neglected tropical disease of public health importance in Latin America, particularly in rural areas. In Floriano, Piauí, Brazil, data on infection prevalence are limited. This cross-sectional population-based study assessed the seroprevalence of *T. cruzi* infection and analyzed sociodemographic factors associated with seropositivity among residents of 41 rural communities in Floriano. A total of 1212 individuals aged over six years were examined, revealing a seroprevalence of 3.2% (39/1212), with positive cases detected in 17 communities. Infection was significantly associated with older age (≥60 years; *p* < 0.0001), low educational level (*p* = 0.0184), retirement status (*p* < 0.0001), and low income (*p* = 0.0505). Logistic regression confirmed age as the strongest determinant of infection (OR = 7.647; 95% CI: 3.741–18.61; *p* < 0.0001), while higher monthly income showed a negative association with infection (OR = 0.17; 95% CI: 0.01–0.82; *p* = 0.086), indicating a trend toward significance. These findings highlight the persistence of *T. cruzi* infection in rural Floriano, predominantly among elderly individuals living in poverty. The identification of previously undiagnosed chronic cases reinforces the urgent need for targeted epidemiological surveillance, early diagnosis, and community-based health education to strengthen local control and prevention strategies.

## 1. Introduction

Chagas disease (CD) is a neglected tropical disease caused by the protozoan *Trypanosoma cruzi* [1]. Transmission occurs primarily through hematophagous triatomine vectors; however, it may also result from blood transfusions, ingestion of contaminated food, organ transplantation, laboratory exposure, and—albeit rarely—sexual contact [2].

From a clinical perspective, CD follows a progressive course encompassing acute and chronic stages. The acute phase appears soon after infection, with high parasitemia and mostly absent or mild symptoms. Without treatment, most patients enter a chronic asymptomatic stage, also referred to as the indeterminate form, characterized by an absence of clinical signs despite persistent infection. This silent stage may last for decades or even throughout life. Over time, approximately 30–40% of infected individuals develop the chronic symptomatic form, primarily affecting the cardiac and digestive systems, and less frequently, the neurological or urinary systems [3,4].

In Brazil, sustained transmission of *T. cruzi* throughout the 20th century has led to a current estimated burden of 1.3 to 3.2 million infected individuals [5]. Due to its epidemiological impact, CD remains to represent a major public health concern in endemic areas and represents emerging challenges in non-endemic regions, largely driven by migratory flows [6,7].

In the state of Piauí, the geographic distribution of CD appears to be influenced by the region’s geomorphology, particularly its mountain ranges and plateaus, which provide ecotopes conducive to the establishment and proliferation of triatomine species [8]. The concentration of reported cases in households located near these environments reinforces this association and highlights the need to strengthen epidemiological surveillance in such areas [9].

A critical barrier to effective disease control is the high prevalence of underdiagnosis and underreporting, a consequence of limited diagnostic infrastructure, low levels of disease awareness, and restricted access to health services [10]. These barriers are particularly pronounced in rural communities, where populations are more frequently exposed to vectors and simultaneously face geographic isolation and logistical constraints that impede healthcare delivery [11].

In this context, Floriano, a municipality in southeastern Piauí, holds particular relevance due to its strategic location and the presence of extensive rural areas with environmental and social conditions favorable to vector-borne transmission [12]. Despite its epidemiological vulnerability, data on the prevalence of *T. cruzi* infection in the region remain scarce. Therefore, conducting serological surveillance in Floriano is essential to fill existing knowledge gaps, support local public health planning, and strengthen disease control strategies in the region.

Thus, serological surveys have been one of the most valuable procedures used as indicators of human infection, serving as a basis for assessing the burden of CD. They also help guide and improve control strategies, justify and motivate the allocation of material, human, and financial resources necessary to ensure full coverage of endemic areas. Accordingly, the present study aimed to perform a serological assessment of Chagas disease in rural communities of Floriano, Piauí, Brazil in order to contribute to improved understanding of the local epidemiological scenario and to inform targeted interventions.

## 2. Materials and Methods

### 2.1. Study Area

The municipality of Floriano, located in the Médio Parnaíba Physiographic Zone in the state of Piauí, Brazil, lies on the right bank of the Parnaíba River, directly across from the city of Barão de Grajaú, in the state of Maranhão. It is situated approximately 240 km from Teresina, the state capital. Floriano has an estimated population of 64,150 inhabitants [13] and serves as a regional hub due to its development in commerce, healthcare, and notably, education.

Floriano is the administrative center of the 10th Regional Health Directorate, which encompasses twelve neighboring municipalities. The municipality’s healthcare infrastructure includes the Tibério Nunes Regional Hospital, an Emergency Care Unit, João Paulo II Hospital, and the Dr. Sebastião Martins Health House. Floriano functions as a referral center for healthcare services in the southern region of Piauí and parts of Maranhão.

In terms of primary healthcare coverage, Floriano has achieved full management of Basic Health Care. The municipality operates 24 health units—seven of which are located in rural zones—along with a polyclinic and specialized centers for the treatment of leprosy and tuberculosis.

Rural communities included in the present study were selected based on a documented history of high triatomine infestation and colonization, or the presence of environmental conditions favorable to vector domiciliation. These included deforested or recently burned landscapes, peridomestic animal enclosures, and human dwellings constructed with mud bricks or adobe. Additional risk factors considered were the accumulation of construction materials—such as tiles, wood, and bricks—in proximity to households, which may serve as shelters for triatomine bugs.

### 2.2. Study Design and Data Collection

This ecological, observational, and descriptive cross-sectional study was carried out among residents of 41 rural communities in Floriano, Piauí, Brazil, where the presence of *Trypanosoma cruzi* vector insects had been reported. Sampling locations were identified with the assistance of Endemic Disease Control Agents (EDCA) and Community Health Workers (CHW), both integrated into Brazil’s Family Health Strategy (FHS). Site selection was based on surveillance data obtained from educational campaigns and vector control programs provided by the municipal epidemiological surveillance department. Fieldwork was conducted between February 2024 and April 2025. Individuals aged six years and older, registered with local primary healthcare units, were eligible for inclusion. Exclusion criteria included individuals who declined participation, persons with special needs, children under six years of age, and minors whose parents or legal guardians did not authorize blood collection. Household visits were performed with the support of community health workers, and participation was voluntary upon written informed consent. A convenience sampling approach was used to collect questionnaire data and blood samples. Structured questionnaires were administered face-to-face by trained interviewers, and blood samples were collected for serological testing and stored at −20 °C until analysis. All laboratory procedures followed standardized protocols, and results were interpreted according to predefined criteria.

### 2.3. Sample Size Calculation

The sample size was determined based on the estimated rural population of 5000 individuals, with an estimated CD prevalence of 3%. Considering a 95% confidence level, 1% precision, the calculated sample size was 1015 individuals to be screened.

### 2.4. Patient Samples and Serological Analysis

For each participant, approximately 8 mL of peripheral blood was collected by venipuncture using a 10 mL disposable syringe and a 25 × 7 mm needle (BD, Juiz de Fora, Brazil). Samples were transferred into CAT Serum Separator Clot Activator tubes (VACUETTE, São Paulo, Brazil). After clot retraction, samples were centrifuged at 3500 RPM for 10 min (Excelsa Baby, FANEM, Guarulhos, Brazil) to obtain the serum, which was then stored at −20 °C until testing. Serum samples were tested for *T. cruzi* antibodies using the rapid test TR Chagas (Bio-Manguinhos, Rio de Janeiro, Brazil) and the BIOLISA Chagas Recombinant (Bioclin, Belo Horizonte, Brazil) according to the manufacturers’ protocols. Briefly, for the rapid test, 5 µL of serum was added to the sample well, followed by three drops of buffer. If no migration occurred within 3 min, the test was discarded; results were read after 15 min. For the BIOELISA, serum samples were added to antigen-coated wells, incubated, washed, and then treated with enzyme-conjugated antibodies and substrate. Absorbance was measured at 450 nm (primary) and 630 nm (reference) using a BIOELISA Reader (Bioclin, Belo Horizonte, Brazil) within 30 min.

Test results were interpreted as follows: Negative—all assays non-reactive; Inconclusive—reactivity in only one assay; and Positive—reactivity in both tests. All positive cases were reported to the local health authorities and referred to as appropriate clinical follow-up.

### 2.5. Geospatial Analysis

Spatial analysis (georeferencing) of positive cases was carried out, enabling the creation of a map in a graphical model using the open-source Geographic Information System (GIS) software (QGIS, version 3.28.2), which displayed the distribution of positive cases found according to place of residence.

### 2.6. Statistical Analysis

Data were analyzed using GraphPad Prism 9. Univariate associations were assessed with Pearson’s chi-square or Fisher’s exact tests (α = 0.05). Variables with *p* ≤ 0.05 were entered into a multivariate logistic regression model. Odds ratios (OR) and 95% confidence intervals (CI) were calculated, and *p* < 0.05 was considered statistically significant.

### 2.7. Ethical Aspects

This study was approved on 16 October 2023, by the Research Ethics Committee of the Federal University of Piauí and the Oswaldo Cruz Foundation/IOC (approval number 6.428.709; CAAE 74095623.6.0000.5214). Participation was entirely voluntary and occurred only after each participant received a detailed explanation of the study’s objectives and procedures. Written informed consent was obtained from all adult participants through the signing of a Free and Informed Consent Form (FICF). For minors (under 18 years of age), participation required the signing of a Free and Informed Assent Form (FIAF), by their parents or legal guardians, in full compliance with national and international established ethical guidelines.

## 3. Results

A total of 1212 individuals from 41 rural communities in Floriano, Piauí, were interviewed, answered the questionnaire and participated in the serological survey. Table 1 summarizes the sociodemographic distribution of studied population according to the analyzed variables (gender, age-group, race/ethnicity, educational level, occupation, Birthplace, monthly income). Sex distribution was relatively balanced with 48.1% (*n* = 583) male and 51.9% (*n* = 629) female participants. Age distribution indicated that the largest proportion of individuals belonged to the 40–59 years group (33.8%), followed by those aged ≥60 years (27.9%), 20–39 years (21.1%), 10–19 years (11.1%), and 0–9 years (5.9%), evidencing a predominance of adults and older adults. Regarding self-reported race/ethnicity, most participants identified as mixed-race (“parda”) (79.6%), followed by Black (14.2%) and White (5.7%). In terms of educational attainment, 46.7% reported completion of elementary education, 22.5% had preschool-level education, 17.6% had completed secondary education, 8.6% were illiterate, and only 4.0% had completed higher education—indicating an overall low educational level within the studied population. Occupational data indicated that 36% were rural workers, followed by retirees (21.95%) employed in general or operational services (16.3%), students (16.2%), homemakers (8.1%), and health professionals (1.5%). Concerning Birthplace, most individuals were born in the municipality of Floriano (79.1%), while 14.9% originated from other municipalities of Piauí, and 5.4% in other Brazilian states. Finally, household income analysis revealed that the majority of participants (79.3%) reported earning up to one minimum wage, 8.8% earned more than this threshold, and 11.9% did not provide income information—underscoring a prevailing condition of socioeconomic vulnerability (Table 1).

Of the total 1212 samples analyzed, 28 (2.3%) presented indeterminate serological results, characterized by reactivity in either ELISA or recombinant test (RT), but not both. Indeterminate cases were not included in the seroprevalence estimates.

**Figure 1:**
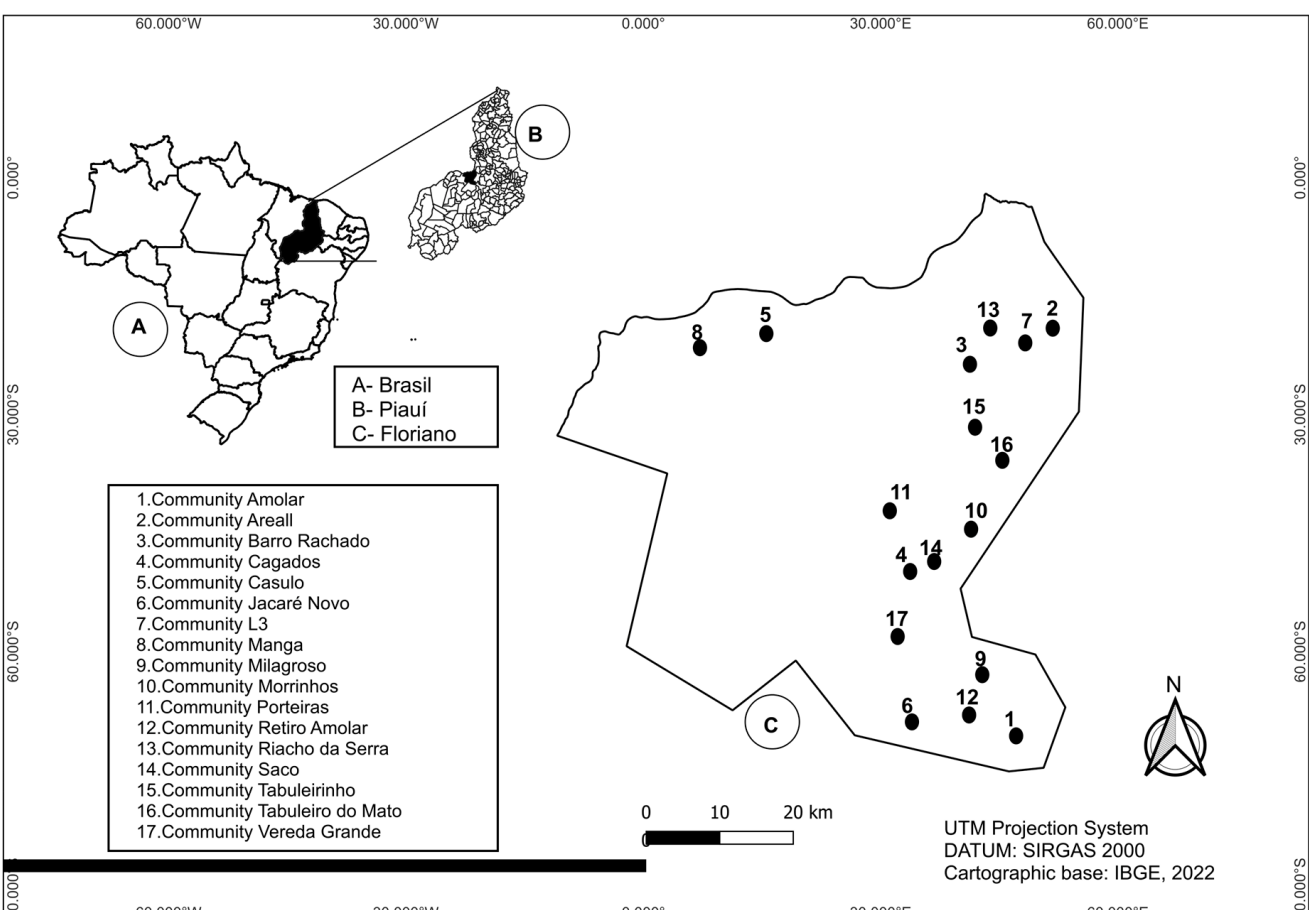
Spatial distribution of positive cases of *Trypanosoma cruzi* infection in the rural communities investigated in the rural area of the Municipality of Floriano, Piauí (*n* = 1212).

Among the 1212 individuals included in the study, 39 (3.2%) tested positive for anti-*T. cruzi* antibodies by both rapid test (RT) and ELISA. These positive cases were distributed across 17 of the 41 surveyed communities, corresponding to 41.5% of the study area (Figure 1).

Analysis of the seroprevalence of *Trypanosoma cruzi* infection in relation to sociodemographic variables (gender, age group, race/ethnicity, education, occupation, birthplace, and monthly income) revealed no significant differences in positivity rates with respect to gender, race/ethnicity, or birthplace. Conversely, statistically significant associations were observed for age group, educational level, occupation, and monthly income (Table 2 and Table 3). Positivity was positively associated with age over 60 years (*p* < 0.0001), low educational attainment (*p* = 0.0184), retirement status (*p* < 0.0001), and monthly income of up to one minimum wage (*p* = 0.0505) (Table 2).

Multivariate logistic regression was performed to assess the association between sociodemographic variables and *Trypanosoma cruzi* seropositivity (Table 3). Age group was strongly and significantly associated with infection, with individuals in older age groups showing a 7.65-fold higher likelihood of seropositivity compared to the younger age groups (OR = 7.647; 95% CI: 3.741–18.61).

Monthly income showed a negative association with seropositivity (coefficient −1.798), indicating that individuals with higher income had lower odds ratio (OR) of infection (OR = 0.1656; 95% CI: 0.009–0.821; *p* = 0.0856), suggesting a trend toward significance. This finding suggests that individuals with higher income may have a lower chance of being infected by *T. cruzi*. In contrast, education level (OR = 1.185; 95% CI: 0.694–1.956; *p* = 0.518) and occupation (OR = 1.231; 95% CI: 0.872–1.782; *p* = 0.2499) were not significantly associated with seropositivity.

These results indicate that age is the primary determinant of *T. cruzi* infection in this rural population, while lower income may contribute to increased risk. Education and occupation do not appear to have a significant effect when adjusted for other variables.

## 4. Discussion

Chagas disease remains a major public health challenge in Brazil, largely due to the substantial burden of undiagnosed and underreported cases within public health services. These factors underscore the critical importance of active surveillance and early detection in vulnerable populations [14].

Despite improvements in housing quality over the past decades, the Northeast region continues to exhibit substantial social vulnerabilities and reports some of the highest rates of housing conditions favorable to colonization by triatomines, the vectors of Chagas disease. Moreover, the limited operational performance of the National Chagas Disease Control Program has hindered the effectiveness of surveillance and control measures in region [15]. This epidemiological profile is observed in rural communities of Piauí, particularly in the semi-arid region, which remain frequently colonized by wild triatomine species. These patterns reflect persistent environmental and structural conditions that facilitate the adaptation of these vectors to domestic environments and sustain the risk of Chagas disease transmission [16].

The municipality of Floriano, although included in this context, lacks updated data on the prevalence of Chagas disease. In the present study, we report, for the first time, the prevalence of Chagas disease in rural communities of Floriano and the sociodemographic vulnerabilities associated with infection risks. Our findings demonstrate a detectable population seropositivity for Chagas disease. The serological survey identified several individuals reactive for anti-*T. cruzi* antibodies, confirming the ongoing epidemiological relevance. The overall seropositivity rate in Floriano was 3.2%, slightly lower than the national survey carried out between 1975 and 1980, where the state of Piauí showed a prevalence of 4.2% [17], but higher than the obtained in the survey of 2002 in Piauí (1.9%) [18]. These findings indicate that, although national control efforts in reducing the prevalence of Chagas disease over time, the infection persists in endemic areas such as Floriano. This persistence may reflect a combination of undiagnosed chronic cases and ongoing residual transmission, underscoring the urgent need for entomological investigations and strengthened active surveillance strategies [19].

Notably, the age-stratified analysis demonstrated that the presence of positive cases among individuals younger than 60 years suggests ongoing transmission of the infection in Floriano. However, most positive cases were observed among older individuals, indicating that the majority of infections were likely acquired in the past and have now progressed to the chronic phase of Chagas disease, consistent with the progressive aging of the population living with the disease [20]. This trend was reinforced by multivariate logistic regression analysis, which confirmed a strong association between increasing age and infection risk, with older individuals showing approximately seven times higher odds of seropositivity compared with younger age groups. These findings corroborate previous studies realized in Brazil and Argentina, where was identified a seropositivity rate of 18.8% among older adults when conducting a serological survey in a prevalence in municipalities in the Northern Minas Gerais State [21], 54.29% in indigenous communities from the south of the Gran Chaco ecoregion in Santa Fe Province, Argentina [22] and 60% in a rural section of Pampa del Indio municipality, Chaco province, Argentina [23].

A study on global trends in Chagas disease conducted by Gómez-Ochoa et al. (2019) [24] showed that the burden of Chagas disease is markedly concentrated in older age groups. This demonstrates that population aging is an important determinant of disease burden, corroborating the findings of this study and highlighting the increasing vulnerability of very elderly individuals. Age is consistently identified as an independent determinant of cardiovascular risk, being strongly associated with comorbid conditions such as hypertension, diabetes, obesity, and dyslipidemia, as well as with increased mortality [25]. In the context of Chagas disease, older individuals exhibit a heightened susceptibility to cardiovascular complications [26]. Consequently, characterizing the prevalence of these age-related patterns and their influence on morbidity and mortality is essential to inform the design of targeted control measures and healthcare strategies, particularly for elderly populations.

In Ceará, similar results were reported by Glass IR et al. (2018) [26], showing that the highest percentage of seropositivity was found in older age groups, which also raises concerns about the onset of organ damage. Likewise, a study conducted in Bahia by Soares et al. (2023) [27] emphasized the need for continuous surveillance monitoring, as it is essential to sustain control efforts and detect any potential reemergence of the disease, given that the positive case identified in their study was an elderly individual who had already developed cardiac complications.

Although not statistically significant in this study, a large proportion of individuals reported rural work as their main occupation. This reinforces the notion that most retirees are former rural workers, reiterating the likelihood of disease chronicity. Similarly, studies by Fidalgo in Ceará, Silva in Piauí, and Colussi in Argentina [22,28,29] emphasized that working in rural areas, particularly in agriculture or subsistence activities, increases the risk of exposure to Chagas disease vectors and, consequently, raises the prevalence of infection in rural populations.

With regard to education, the present study demonstrated statistically significant association between seropositivity and having completed only primary education. Corroborating these findings, the 2024 Brazilian Epidemiological Bulletin reported that individuals with low educational attainment present the highest prevalence of Chagas disease infection. This finding indicates that the lower the level of education, the higher the risk of illness, making health education initiatives one of the key strategies to be developed. Furthermore, Ochoa-Diaz et al. (2024) [30], in their study conducted in the Caribbean, also highlighted the prevalence of seropositivity among rural residents with low educational attainment, specifically primary education, which is consistent with the results presented here. In Ceará, Fidalgo et al. (2021), [28] also emphasized the need to develop health actions for the population, including general aspects of Chagas disease and the ecological characteristics of triatomines. This need stems from the low educational level of the seropositive individuals identified in their survey conducted in the municipality of Quixeré.

Income was also a significant determinant, with higher seropositivity observed among individuals reporting earning up to one minimum wage. This reflects the influence of socioeconomic factors on disease persistence, as they directly affect living conditions, quality of life, and interaction with the environment [31]. Poverty, expressed as low income, is one of the main factors associated with the onset of diseases, since human behavior plays a decisive role in shaping how zoonotic diseases emerge over time.

In this context, several authors have demonstrated that individuals with low socioeconomic status are at greater risk of acquiring Chagas disease, primarily due to precarious housing conditions and inadequate dietary practices [32,33]. Moreover, the Pan American Health Organization (PAHO) emphasizes that the main risk factors for Chagas disease include living in substandard or poorly constructed housing, residing in areas characterized by economic deprivation or socioeconomic instability, and having limited access to resources. In this regard, insufficient income is frequently identified as one of the key social determinants that perpetuate vulnerability to the disease [34].

Thus, there is a clear need for active and effective surveillance, since most cases remain hidden due to the lack of diagnosis or active surveillance systems. Likewise, the need for continuous health education for the population is reinforced, particularly regarding household hygiene and housing improvements [35,36].

## 5. Conclusions

Our findings confirm the persistence of Chagas disease in the municipality of Floriano and reinforce the value of serological surveys as effective tools for timely and reliable case detection. The study highlights the ongoing transmission of *T. cruzi* in vulnerable rural settings and underscores the influence of social and demographic factors—such as aging, limited educational attainment, and economic constraints—on the epidemiological profile of the disease.

The identification of specific communities with confirmed seropositivity indicates that *T. cruzi* transmission is not random but potentially shaped by local social and environmental determinants. This pattern emphasizes the need for targeted surveillance and control measures in areas with concentrated cases, as well as the consideration of local ecological and socioeconomic factors that may affect infection risk.

These findings provide critical evidence to support the design and implementation of focused active case-finding initiatives and tailored preventive interventions. Furthermore, the results underscore the importance of sustained and strengthened epidemiological surveillance, integrated with the development of comprehensive control strategies. Ensuring equitable access to diagnostic services for rural communities of Floriano should be regarded as a public health priority, essential for reducing disease burden and interrupting potential transmission.

## Figures and Tables

**Table 1:** Sociodemographic Profile of the Population Included in the Serological Survey in Rural Communities of Floriano, Piauí (*n* = 1212).

Variables	N°	%
**Gender**		
Male	583	48.10
Female	629	51.90
**Age-group**		
0–9	72	5.94
10–19	135	11.14
20–39	256	21.12
40–59	410	33.83
≥60	339	27.89
**Race/Ethnicity**		
White	70	1.32
Mixed-race (Parda)	966	79.62
Black	172	14.19
Asian	3	0.25
Indigenous	1	0.08
**Educational level**		
Illiterate	105	8.63
Preschool education	275	22.53
Elementary education	570	46.71
High school	213	17.60
Higher education	49	3.95
**Occupation**		
Rural worker	436	35.97
Student	196	16.17
Homemaker	98	8.09
Health professional	18	1.49
General/Operational services	198	16.34
Retired	266	21.95
**Birthplace**		
Floriano Municipality	959	79.13
Other municipalities in Piauí	180	14.85
Other states	65	5.36
Not informed	08	0
**Monthly income**		
Up to 1 minimum wage	961	79.29
Above 1 minimum wage	107	8.75
Not informed	144	11.88

**Table 2:** Correlation between seroprevalence of *Trypanosoma cruzi* infection and analyzed variables in the rural population of the municipality of Floriano, Piauí (*n* = 1212).

Variables	Total	Positive Cases		*p*-Value
		N°	%	
**Gender**				
Male	583	13	2.22	0.442
Female	629	26	4.13	
**Age-group**				
<20	214	0	0.00	<0.0001 ^#^
21–40	268	1	0.37	
41–60	415	5	1.20	
>60	315	33	10.47	
**Race/Ethnicity**				
White	70	4	5.71	0.7406
Mixed-race (Parda)	966	31	3.21	
Black	172	4	2.33	
Asian	3	0	0.00	
Indigenous	1	0	0	
**Educational level**				
Illiterate	380	21	5.53	0.0184 ^#^
Preschool education	570	14	2.46	
Elementary education	213	3	1.41	
High school	49	1	2.04	
**Occupation**				
Rural worker	433	6	1.39	<0.0001 ^#^
Health professional	20	1	5.00	
Retired	266	27	10.15	
others	493	5	1.01	
**Birthplace**				
Floriano Municipality	959	31	3.23	0.6563
Other municipalities in Piauí	180	7	3.89	
Other states	65	1	1.54	
**Monthly income**				
Up to 1 minimum wage	961	37	3.85	0.0505 ^#^
Above 1 minimum wage	107	2	1,87	
Not informed	144	0	0	

^#^: Significant *p*-value.

**Table 3:** Multivariate logistic regression analysis of sociodemographic factors associated with *Trypanosoma cruzi* seropositivity in the rural population of Floriano, Piauí, Brazil.

Variables	Coefficient	SE	DF	*p*-Value	Odds Ratio (CI 95%)
Educational level	0.1697	0.2625	4	0.518	1.185 (0.694–1.956)
Occupation	0.2077	0.1805	4	0.2499	1.231 (0.872–1.782)
Monthly income	−1.798	1.046	4	0.0856	0.1656 (0.009–0.821)
Age-group	2.034	0.4043	4	<0.0001 ^#^	7.647 (3.741 to 18.61)

SE: Standard Error; DF: Degrees of Freedom, CI: Confidence Interval; ^#^: Significant *p*-value.

## Data Availability

The data and materials are available from researchers at Protozoology Laboratory/IOC/FIOCRUZ. The data presented in this study are available on request from the corresponding author. The data is not publicly available due to ethical restrictions.
